# Some Thoughts of Medical Education

**Published:** 1958-01

**Authors:** C. Bruce Perry


### SOME THOUGHTS OF MEDICAL EDUCATION

*Presidential Address delivered to the Bristol Medico-Chirurgical Society 9th October, 1957*

BY

C. BRUCE PERRY, M.D., F.R.C.P.

Despite the fact that Sir Robert Hutchinson is said to have referred to Medical Education as "that most boring subject", I have succumbed to the temptation to make it the subject of this address. I have done so for three reasons. Firstly because I have naturally been interested by, and involved in, medical education for many years and some two years ago the trustees of the Rockefeller Foundation gave me the opportunity of spending three months studying Medical Education in the United States. My second reason is that for some time there has been a growing sense of dissatisfaction with our present methods of medical education, which has resulted in a spate of lectures and conferences. In fact, an American observer remarked some years ago that in the past ten years no country has produced so many wise reports on the improvement of medical education as Great Britain, and no country has done so little about it. The third reason that makes me feel that the present moment is a very opportune one at which to consider afresh the whole problem of medical education is the great changes which might have followed the new Medical Act of 1950 and the fact that new recommendations have this year been issued by the General Medical Council.

The Medical Act of 1950 means that no longer does a degree or diploma entitle its holder to practice medicine unless and until he or she has carried out practical work in hospital under supervision for twelve months and such work has been certified as satisfactory.

In an attempt to safeguard the standards of professional competence the General Medical Council has from time to time made recommendations to the Medical Schools that certain subjects should be taught in a certain order for certain minimum periods. It is true that the Council has always taken care to point out that these are only recommendations, but in point of fact they have come to have the force of regulations. Further, their recommendations have been added to with each new issue so that in 1947 the minimum requirements could hardly be completed, if carried out conscientiously, before graduation. Now, however, the policy has been reversed and Universities are being allowed freedom in which to experiment in the nature and content of the medical course.

*Education and the Curriculum*

The first question which I ask myself is whether we are justified in calling what we give our present students a medical *education* at all. In fact I sometimes wonder when I hear discussions about the need of technical institutes for technological training how soon my colleagues in the other faculties in the University will discern that the medical faculty is little, if at all, better than a technical school and throw us out of the University in disgrace. We were told at our last meeting that in Russia, where after the revolution more doctors were, and still are, required in very large numbers, the medical schools are quite outside the Universities and are now virtually technical colleges.

Charles Newman in his recent review of medical education in the nineteenth century has obviously had similar doubts and has apparently come to the conclusion that about 1900 medical education in this country was replaced by the "medical curriculum".[1]

The first doubts about the efficiency of medical education as we know it to-day were perhaps expressed by Osler[2] who, in 1899, in an address at McGill said: "Undoubtedly the student tries to learn too much and we teachers try to teach him too much—neither perhaps with great success. The existing evils result by neglect on the part of the teacher, student and examiner of the great fundamental principles laid down by Plato—that education is a lifelong process in which the student can only make a beginning in his college course". However, the first real shock came from the famous report of Abraham Flexner[3] in 1925, which, with the earlier report on medical schools in the United States, did so much for medical education in the United States and probably all over the world. In this report Flexner said, "Medical curricula the world over contain too many subjects as well as too much material. The burden would be heavy enough if it were confined to the larger original units, but within the last fifty years one speciality after another has been split off, erected into a professorship, made a subject of special teaching and finally won a place on the examination list". That was over 30 years ago. Think of the new subjects that have been invented since. He went on to remind us that "men become educated by steeping themselves thoroughly in a few subjects and not by nibbling at many."

Twenty-five years ago, Lord Moran[4] in a paper, aptly entitled "The Student in Irons", wrote "the student caught up in the clinical years in a blind rush to meet the tyranny of signing up, is hurried out of thought. He must be driven into different pens for every hour of the day in the hope that one day he may recognize a disease because he has seen it before, as a sleepy traveller marks his destination by night".

In 1944 the Goodenough Report[5] which spread encouragement and hope through the medical schools of the country restated the need for re-organization of the medical schools and teaching hospitals but prophesied that any such steps would be largely barren of any result unless the present overloading of the curriculum was remedied. The load of work upon undergraduate students, it said, must be lightened by the elimination of all that is redundant or belongs to the province of the specialist. "This reform" it went on, "is so urgent that its accomplishment might well be a condition, to be satisfied before any increase is made in Exchequer grants in aid of undergraduate medical education". As far as I know no university refused increased Exchequer grants, but only one, and that in the last twelve months, has done anything to reduce the content of the medical curriculum. As Osler[6] said, over 40 years ago, "The pity of it all is that we should have made an intolerable burden of the study of one of the most attractive of the professions".

In the face of such criticism how can we claim that our crowded curriculum is a medical education. For to quote Professor Melville Arnott[7] the fundamental object of medical education, indeed of all education, is to develop the human mind as an instrument of observation, deductive and inductive reasoning and rational expression in word and action, whereas in our medical curriculum to-day the tendency is to require the student "to assimilate a series of dicta, without enquiring into their validity or necessarily considering the evidence on which they are based". This is, of course, a feature, as Sir George Pickering[8] has reminded us, which is characteristic of instruction as opposed to education. Pickering goes on, "It is a process which does not necessarily inform the mind of the recipient and may actually do harm by suppressing curiosity, insight and the capacity to learn from experience". Now it is evident that the present overcrowding of the curriculum can have only one result, namely that education gives place to instruction.

Not only has the curriculum become crowded but the length of the course has also been increased. This lengthening of the period of study has extended into the post-graduate years and recently it has been calculated that if the changes which have operated over the past hundred years continue it will, in Boston by 1970, take eighteen years to produce a surgeon.[9] I am not sure that we are not ahead of the United States in this matter as it seems to take nearly as long as that in England now.

*How has all this come about?*

Medical training starting as an apprenticeship was at first quite unorganized. In fact, before 1800 anyone could practice or write about medicine. (John Wesley, for instance, wrote a book on *Primitive Physic: or An Easy and Natural Method of Curing Most Diseases*. This was so popular that by 1820 there had been 29 editions. Although written by a cleric, who might be regarded as a "quack", he advised the use of official preparations, as shewn by the last paragraph of the book: "I advise all, in or near London, to buy their medicines at the Apothecaries' Hall. There they are sure to have them good".) However, at the end of the eighteenth century, so-called medical schools sprang up, which were really schools of anatomy and these developed tenuous associations with hospitals in which it was beginning to be the fashion for some of the apprentices to work. It is perhaps this priority which has given anatomy the great influence and prestige which it still has in the medical curriculum. Gradually other studies were included, the first of which was the "Institutes of Medicine" introduced by the famous Boerhaave in 1701 at Leyden.[10] This at first included physics, chemistry, biology and physiology, and to these pathology was later added. Boerhaave really originated the present plan of the medical course divided into pre-clinical and clinical studies, although, of course, in some countries, particularly perhaps in France, there is still no division between the two, the student being introduced to the wards or out-patients from his first day as a medical student.

The medical schools were not in their origin university schools—rather the reverse, as in the provinces most "Redbrick" Universities were developed in association with pre-existing medical schools having associations with the local hospitals and in which the teaching had become organized and supervised as a result of the Medical Act of 1858. Whereas in the case of the London schools they developed and remained part of the hospitals and even now are only gradually being absorbed into the university. During the past 100 years, several factors have contributed to the present appalling state of the curriculum. One is the fact that until the Medical Act of 1950 a degree or licence conferred the right to practice. Hence great anxiety was felt that the curriculum and final examination should ensure that the graduate was "safe" to practice. As each speciality developed, as Flexner said, it fought for its share of the student's time and its place in the final examination. This effect was augmented by the regular appearance of the recommendations of the General Medical Council, to which reference has already been made. Each one of these (until the one issued this year) added further subjects to be studied and usually recommended that previously listed subjects should be studied for a longer time. It is perhaps noteworthy that the only response the General Medical Council made to the plea of the Goodenough Report for a reduction in the curriculum was to increase the detail of its recommendations in nearly every subject. In medicine a requirement for a month's residence in hospital was added, and three subjects, hitherto included in medicine, were exalted into major subjects in their own right with separate departments and examinations.

The result of all this has been that the curriculum has become more and more crowded with compulsory lectures and class work so that the student no longer has any time in which to think about the subjects studied or to pursue individual lines of enquiry.

When Mr. T. F. Edgeworth was a student at the Bristol Medical School (in Old Park) and the Bristol Royal Infirmary in the early 1850's he had to be "signed up" for seven subjects (practical pharmacy, anatomy, physiology, surgery, physic, materia medica and midwifery) in order to enter for the final examinations for the membership of the Royal College of Surgeons. Compare this with our students schedule to-day, in which signatures are required in the clinical part of the course for 25 courses of lectures or demonstrations and 18 clinical appointments.

*Need for "free-time"*

It is illuminating to compare the time-table of our students in their third year when they have 28 hours of set work a week, with that of a third year science student reading Geology, which occupies 19 hours a week, or that of a third year arts student in the English Honours School, with 10 hours of set work. Yet this overcrowding of the time-table is not unavoidable as can be seen by considering similar time-tables for students in for instance the Johns Hopkins Medical School where Tuesday and Friday afternoons and the whole of Saturday are "free". In fact in nearly all the thirteen medical schools that I visited in the United States the great importance of "free time" was clearly recognized. In the great experiment at Western Reserve University, where the whole medical course has been re-designed afresh, one of the four great guiding principles which the planners adopted at the start was that every student should have 1½ days a week free.

### EFFECTS OF FINAL EXAMINATIONS

Another factor which makes the student try to become a sponge absorbing so-called "facts" is, as Osler realized, the content and nature of our final examinations. We all know that it is impossible for anyone to learn all about medicine or any other branch of the curriculum in 20 years, let alone five, yet the student spends his final years with rapidly increasing anxiety that in his finals he will in fact be asked something he does not know. Thus the rush to complete appointments and get signed up in the fourth and fifth years is replaced in the sixth year by frantic efforts to commit to memory more and more "important" facts. One of the aspects of this, which would be laughable were it not so tragic, is the undoubted fact that many of the "facts" of to-day will be discarded in ten years time. And none of us know which.

What has been the purpose of this final examination which exerts such an influence over the life and behaviour of the student? First of all I suppose it was designed in an attempt to ensure that the newly qualified practitioner would be "safe to let loose on the public". I wonder if it has really ever achieved this.

#### *Pre-registration posts*

We all know that for a long time we have always preferred newly qualified practitioners to undertake further training as housemen in hospitals and, or, as assistants in practice before embarking on independent practice. In any case, as I have said, the Medical Act of 1950 should have removed any anxiety on this score. Unfortunately, there is felt in many quarters grave doubts whether the pre-registration year is really functioning as it should. There is no doubt that in many of the recognized posts the pre-registration houseman has far too much to do—some have to look after as many as 80 beds—far too little time and opportunity for furthering his education by reading journals and discussions with his colleagues, and far too little supervision by his chiefs. The difficulty of course is that when the Act was implemented it was assumed that what was needed as a pre-registration post was the old fashioned resident appointment. The hospitals wanted their posts recognized as it was the only way in which they were able to obtain junior medical staff. The universities and licensing bodies were anxious to recognize as many posts as possible as if there were not enough posts recognized then pre-registration practitioners would be out of work through inability to obtain the necessary appointments. (This has happened in some cases). The only way to make this pre-registration year really educational would have been to double the posts in certain first-class hospitals so that the pre-registration officer would be in a stimulating atmosphere and have charge of no more than 20 to 25 beds as the Goodenough Report originally recommended. It is, of course, at the end of this pre-registration year that the practitioner should be declared safe to practice and to this end every licensing body receives a report on the work of the graduates and it is on these reports, if satisfactory, that admission to the Register is recommended. I wonder how many graduates have been required to do more than the minimum two-pre-registration posts because their chiefs and supervisors have not considered them satisfactory.

#### *Are Final Examinations necessary?*

To go back to the final examinations. It is claimed that they have maintained standards and this may be so in that since most examiners examine at some time or another for several examining bodies the final examination is much the same wherever conducted. Another function that has been claimed for the final examination is that it is an important stimulus to work and that it not only makes the student work but influences the subjects he studies. It is well known that students read past examination questions very carefully and thus if a topic figures frequently in examinations it will surely receive a fair amount of attention from the students. It may be true that the students do need a stimulus to make them work but there is little doubt that the present final examinations make them work in the wrong way. Instead of trying to understand a subject the student with the dread of the ordeal of the final examination hanging over him is driven into a frenzied effort to memorize what he hopes will, in the examination, be useful facts. This of course is cramming and not education.

The medical schools of the United States have shown that you can make use of examinations as an incentive to work and yet abolish the final examination as we know it. In the pre-clinical years the American student is subjected to terminal or more frequent examinations to make him work. In the clinical years there may be a *viva voce* or small examination at the end of each phase such as the three months' medical clerking but at the end of each such period his work and progress is carefully considered by all those who have taught him and he is given a "grade". If this is unsatisfactory he is required to repeat this part of the course. But there is no final examination as we know it and the degree of M.D. is given largely on the gradings awarded all through the course. No American student in the clinical years ever thinks of the possibility of failing to graduate. In fact, they all fix up their internships, which are usually held in another hospital from that in which they have been students, months before they are due to graduate. Thus the final months are free from anxiety either, as to graduation, or, as to if and where they will get a house post, and the student can devote his time and energy to real study in any branch of medicine in which he feels particularly interested. In fact he can begin to educate himself.

The final examination and the high fail rate in this examination is one of the features which should show us clearly that all is not well with medical education. How is it really possible to defend a system in which after a very careful selection the picked student is taught for five or six years, at the end of which time on the average about 25 per cent fail to pass the final examination conducted by those who have selected and taught them. (And in some universities the fail rate has in the not very distant past been as high as 40 per cent).

The final examination has recently come under severe criticism from another aspect and it is alleged that in point of fact it is inefficient as a test of the student's knowledge and ability. Professor Bull[11] of Belfast has re-marked some of the orthodox essay type of examination papers after a lapse of time and found that there was considerable variation in the assessment and marking on the two occasions. Criticism such as this has aroused fresh interest in the short factual type of examination question and even in the multiple choice type of examination so popular in the State qualifying examination in the United States where all the candidate has to do is ring a number or underline the correct word. Such examination papers are of course extremely difficult to set but the marking is automatic and can if need be, be performed by an electronic machine.

### AMERICAN MEDICAL EDUCATION

I should perhaps explain that while there is no final degree examination in the majority of the medical schools in the United States each individual state has its own requirements for registration for practice and most require the candidate to sit a fairly simple examination. This is usually taken near the end of the compulsory twelve months' internship which roughly corresponds with our pre-registration year. It seems to be rare for any ordinary candidate to fail.

### *Students and Staff*

There is another striking difference between the medical schools of the United

States and Great Britain and that is that staff/student relationship. In the States the student is in no fear or awe of his teacher and is encouraged to discuss cases and problems freely with him. There are no strange delusions about the infallibility of professors for in the grand rounds and conferences which are held daily the student may present the case, but he then hears it discussed fully by all members of the staff and even by his student colleagues and he rapidly learns that there are many different view points on the majority of problems.

*Students' responsibility for patients*

This leads me to another matter which sometimes causes me concern and that is that our students seem no longer to have any sense of responsibility for the study or care of their patients. Twenty years ago the medical clerk was made to feel that he was an essential member of the team caring for the patient. This sense of responsibility was of course a great spur and stimulus to him. Certainly since the war this has largely disappeared. I do not think it is because of the increased number of junior staff, such as registrars and the like, since in the States the staff in a teaching hospital is far greater than anything we have ever dreamt of. It may, I think, be due to the fact that as part of their training we now require the residents to write notes on the patients, and the students feel that their own note taking is therefore superfluous and a waste of time. In the States the student responsibility is carried to great lengths. In some out-patient departments the patient is first seen by a student, who has his own consulting room with his name (Dr. Smith) on the door. He spends 1½ to 2 hours with the patient taking a full history, making a complete clinical examination and any pathological studies he thinks indicated and then presents the "worked up" case to his teacher. Then follows a consultation on the diagnosis, any further studies that might help, and treatment. The student then arranges the further study or prescribes the treatment. Of course, few of the cases in the out-patient department are referred and out-patients are only to a slight extent consultative.

*Organization of Teaching*

The free participation by the student in the discussion of a patient is facilitated by the organization of the teaching. For years we in Great Britain have been told that in order to impress the student with the importance of, say, pathology or preventive medicine these subjects must be taught throughout the course. We have tried to do this and have spread the formal teaching over perhaps three years. The result is that when the clinical clerk in the fourth year meets a patient with mitral stenosis he has no idea of what this is, or what it looks like, because he is going to be taught about the pathology of heart disease next year. So, too, in order to make the student continually work at pharmacology, we put the examinations in pharmacology as late in the course as we dare, with the result that when in the fifth year the student is doing obstetrics and should in the evenings be reading about the various types of labour, instead, so great is his anxiety about the examination next month he spends all his time learning about the pharmacology of hexamethonium. By contrast, in the States the teaching in pre-clinical subjects is often arranged in "blocks" so that for say, ten weeks the whole time is devoted to pathology which is then completed. As a result, when the student meets his first case as a clinical clerk, he may never have handled a patient before but he knows theoretically all about the aetiology and pathology of the disease and about the pharmacology of the drugs that may be used and has had instruction in the symptoms and signs. It is clear therefore why he is able to discuss the case with his teacher intelligently.

Another aspect of medical education which has received great attention in the United States is the problem of "integration". I have already mentioned how our students may hear about the pathology of a disease one year and the aetiology next year and so on. Is it possible or desirable so to organize the course that on the day he first hears about suppuration the student also learns about the staphylococcus, sees a patient with a boil and learns about the surgical technique of opening it and the pharmacology and methods of administration of penicillin. It is possible but clearly the organization necessary to achieve such as an arrangement as I have outlined makes enormous demands on teacher time and this has to be repeated every day. It has been suggested that it is not only difficult but undesirable and that the best place for integration to take place is in the student's own mind. On the whole I rather agree with this view. However, it has one great dvantage and that is that the barriers between departments are broken down. All teachers must know when and how their colleagues are teaching their subject. At Western Reserve which, as I have already mentioned, has designed a new medical course, integration has been one of the main principles. As a result, there are no departmental class rooms or laboratories. There is a laboratory class room for each year where every student has his own desk, laboratory bench and cupboard and where he can work at any time of the day or night. The department and the teacher come to the student who occupies his own desk whether he is at a bacteriology lecture or a practical physiology class. The course is so designed that at the same time that the student is taught about the anatomy—gross and microscopic—of the liver, he learns about its physiology, the pathological changes that may affect it, and the clinical features of the disease so produced. But in order to achieve this a special grant from the Commonwealth foundation was necessary so that the teaching staff could be enormously increased. Even now the teachers have to spend much time in conferences planning the timing of the instruction. It is a fascinating experiment but one I feel which does not justify the cost.

### *Functions of a University*

Up to date I have failed in my efforts to make my students discuss cases and argue with me in the way the American student does. Yet surely this is the essential basis of true education and the main object of a University: described by Sir Edward Appleton[12] in his Reith Lectures as "a place of intellectual roads and bridges: it provides the student with opportunities for contact with other minds and other disciplines". Sir Clifford Allbutt[13] once stated that the function of a University "is not qualification for the practice of any art or trade, but is a training of the mind, a formation of habits of study, of insight, of easy handling of ideas and of methodical research . . ."

In the paper on the "Student in Irons" to which I have already referred, Lord Moran said of English medical training: "The one purpose of the student's years is, it seems, not to train and test habits of thought but to collect and store a set of facts, as squirrels hoard the nuts on which they hibernate. These facts are his capital . . . Presently it shrinks and he must needs replace it by a store of wordly wisdom built upon a growing discernment of the ways of human nature, so that soon he is as cunning in the management of men as he is helpless in the control of their diseases . . . The little he knows is soon antiquated, and he cannot keep up with a rapidly changing science, for we have so contrived to educate him that he cannot educate himself".

We all hope that this is an exaggeration but we all also know in our hearts that there is an unpleasantly large element of truth in it.

We must recognize that medicine is indeed a rapidly changing science. Further a medical graduate now has open to him a widely varied choice of work. He can become a medical administrator, an expert in preventive medicine, a pure research worker, a teacher in the basic sciences, a laboratory worker, a consultant, a specialist in an ever narrowing clinical field or a general practitioner. Once upon a time nearly every medical graduate practiced more or less as a general practitioner looking after individual patients. Now probably less than half the medical graduates ultimately work in general practice. What is the sense in trying to ensure that all these people are "safe to practice"? Surely we should realize the position and our aim should be to graduate men and women with the essential basic background, imbued with the spirit of study and so educated that they will go on learning by themselves, whatever branch they take up and remain students all their lives. To borrow the zoological metaphor used by Professor Harris[14] at the last Home Universities Conference, we should try to turn out totipotential free swimming larvae capable of independent existence and development, with no further formal instruction, into any and every type of medical man.

*Teachers of Medicine*

You will have noticed that up to now I have not discussed the question of the teacher or the taught. The selection of medical students is an extremely difficult one, but as on graduation there is such a wide range of openings available to the medical graduate it seems to me that we should accept a wide variety of types of student and accept them for varying reasons. The things that are essential are good intellectual equipment and a capacity for and willingness to perform hard work. Whilst there has, ever since the war, been a very rigorous selection of medical students, there has, as far as I know, never been any selection of medical teachers—at least on the clinical side—on the basis of their ability to teach. In fact this seems often to have been the last thing that was considered when most of us were appointed. We were appointed to the teaching hospital, I think, mainly because it was hoped that we would make good physicians or surgeons and to a lesser extent because we had turned out a modicum of original work.

It is, of course, extremely difficult to know what makes a good teacher and I am not at all sure that the student is necessarily the best judge of that. Under our present system the average student rates highest the teacher who can concisely give him ten causes of ascites. But to me this is the very worst sort of teaching which stifles thought and encourages blind memorization. In some of the schools I visited in the United States I found that the head of the department of medicine received from every student at the end of every term an appraisal of his teachers. If any one teacher consistently received bad reports from the students he was warned and if the bad reports persisted he was not asked to teach next year.

At Illinois medical school lectures have recently been attended not only by students but also by staff colleagues of the lecturer[15] both of whom have recorded their opinion of the lecturer. There was quite a difference in some cases between staff and student assessment. (Incidentally in this study 80 per cent of the students stated that they considered lectures a waste of time.)

You will have seen from what I have said already that I do not think the teacher as a teacher very important. If the essential thing is for us to teach the student to learn for himself then the teacher's main function is to make opportunities and to stimulate interest and enquiry.

I must apologize if I have told you at some length about my impressions of medical education as I saw it in the United States, but it was a stimulating experience. I do not want you to go away thinking that in my view everything is right in American medical education but I do think that we have much to learn from it. I did come to the conclusion that however short the American medical graduate may be in his store of facts as compared with his British counterpart on graduation he is a far more exciting person to meet and talk to and is far more alive to the growing parts of medical knowledge and to the ways in which medicine may develop. He has learnt to learn.

To quote Osler[6] once more:

"The conclusion of the matter is the student needs more time for quiet study, fewer classes, fewer lectures, and, above all, the incubus of examinations should be lifted from his soul. To replace the Chinese by the Greek spirit would enable him to seek knowledge for itself, without thought of the end, tested and taught day by day, the pupil and teacher working together on the same lines, only one a little ahead of the other. This is the ideal to which we should move."

### REFERENCES

1 Newman, C. (1957). *The Evolution of Medical Education in the Nineteneth Century*. Oxford.

2 Osler, W. (1899). *Montreal Med. Journ.*, **27**, 823.

3 Flexner, A. (1925). *Medical Education.* New York.

[4] Wilson, C. M. (1932). *B.M.J.*, **i**, 485.

[5] *Report of the Inter-departmental Committee on Medical Schools.* H.M.S.O. 1944. London.

[6] Osler, W. (1913). *B.M.J.*, **ii**, 946.

[7] Arnott, W. M. (1955). *Lancet*, **ii**, 783.

[8] Pickering, G. (1955). *Lancet*, **ii**, 1149.

[9] Cope, O. (1957). *J.A.M.A.*, **164**, 544.

[10] Snapper, I. (1955). *Meditations on Medicine and Medical Education.* New York.

[11] Bull, G. M. (1956). *Lancet*, **ii**, 368.

[12] Appleton, E. (1956). *Science and the Nation.* The Reith Lectures. Edinburgh.

[13] Allbutt, T. C. (1906). *On Professional Education with special reference to Medicine.* London.

[14] Harris, J. E. (1956). *Home Universities Conference. Report of Proceedings.*

[15] Samter, M., Lepper, M. M., and Montgomery, M. M. (1957). *Ann. Int. Med.*, **46**, 468.

